# Supplementary material for: Use of the Chainchecker application: Uganda’s experience during the 2022 Sudan Virus Disease outbreak
Source: PLOS Glob Public Health. 2025 Apr 21;5(4):e0004352. doi: 10.1371/journal.pgph.0004352 (PMC12011214; doi:10.1371/journal.pgph.0004352)
Supplement: S1 Table — (DOCX) [file pgph.0004352.s001.docx]

| S1 Table. Updates made to Chainchecker capabilities since the initial R Shiny version | |
| --- | --- |
| Theme | Update |
| Dataset management | - Allow users to map variables in their dataset to Chainchecker variables, thus eliminating the needs for user datasets to conform to a particular structure - Allow users to save mappings to quickly re-import data sets - Ability to edit user datasets within the Chainchecker application once they have been uploaded. The changes in the edited datasets are immediately available for analysis - Ability to export edited user datasets - Ability to export chains of disease transmission as various image formats - Ability to modify settings for the application, to include disease, language, incubation period - Data validation window that flags possible data anomalies in the uploaded dataset - Ability to add new diseases and default parameters |
| Visualization of chains of transmission | - Ability to zoom in and out - Users can use their mouse to hover over a node to see additional details about the individual - User can select which variables in their dataset they would like to appear in the hover-over text box - Summary statistics are generated for each chain of transmission that describes the number of plotted cases and the number of cases without epidemiologic links - Ability to choose the icon representing the case/node - Ability to filter displayed cases based on variables in the uploaded dataset and time period - Ability to overlay data layers to include:   - Healthcare facility visits   - Isolation date   - Date of death/recovery   - Other user-defined variables - Ability to display relationships between individuals - Ability to overlay the time period when the individual was likely exposed to the virus - Graph settings   - Show / hide grid lines - Export as png or svg, show full graph or specific view of graph - Ability to toggle an algorithm to show cases that are potential instances of nosocomial transmission - Ability to toggle overlay to color-code cases based on their ‘biological feasibility’ – does the exposure windows / infectious period make sense, does genetic distance make sense, etc.? |
| Visualization of Healthcare Visits | - Ability to view all patients who transited through a particular healthcare facility - For a selected individual, visualize all the healthcare facilities he/she visited - Flag potential instances of nosocomial transmission |
| Visualization of genetic sequencing data | - Ability to overlay genetic sequencing data on top of the epidemiologic data - Ability to identify related and unrelated cases based on genetic data. - Ability to modify viral substitution rate   Ability to overlay the timespan for observing at least one mutation based on viral substitution rates. |
