# Supplementary material for: Use of the Chainchecker application: Uganda’s experience during the 2022 Sudan Virus Disease outbreak
Source: PLOS Glob Public Health. 2025 Apr 21;5(4):e0004352. doi: 10.1371/journal.pgph.0004352 (PMC12011214; doi:10.1371/journal.pgph.0004352)
Supplement: Attachment — Submitted filename: point-by-point response.docx [file pgph.0004352.s002.docx]

Dear Editor and reviewers, thank you for your consideration of this manuscript ‘Use of the Chainchecker application: Uganda’s experience during the 2022 Ebola Disease outbreak.’

Please find the point-by-point responses to the comments.

**Comment 1**
Where possible, we encourage authors to provide relevant data underlying their research with their submission in order to facilitate editorial and peer review, even if the data may be restricted from publication or made private by a data repository prior to article acceptance. Many repositories permit private access for review purposes, and have policies for public release at publication. For data concerning human research subjects, please anonymize the data as appropriate and follow pertinent legal and ethical protocols prior to sharing with editors and reviewers. If you are unable to share any pertinent data at the time of editorial and peer review, please kindly let the journal office know.

**Response:**

The datasets upon which our findings are based belong to the Uganda Ministry of Health and Uganda Public Health Fellowship Program. For confidentiality reasons, the datasets are not publicly available. The de-identified datasets can however be availed upon reasonable request (Investigators whose proposed use of the data has been approved by an independent review committee identified for this purpose from the corresponding author with permission from the Uganda Ministry of Health and Uganda Public Health Fellowship Program after article publication. This has been updated on lines 264-271, page 13

**Comment 2**

Please ensure that the Author List in your manuscript file matches the Author List in the online submission form, including initials and order. Currently, author is  Mercy Wendy Wanyana listed in the online submission form but Mercy W. Wanyana from the manuscript.

**Response.** The author name in the system has been updated to Mercy W. Wanyana

**Comment 3**

Please complete all items on the Human Participants Research Checklist that are relevant for your submission, by following this link: <http://journals.plos.org/plosone/s/file?id=dc11/PLOSOne_Human_Subjects_Research_Checklist.docx>. There may be overlap between the checklist items and other queries listed here; please address any duplicated queries both in your response email and on the checklist itself. Upload the completed Human Participants Research checklist as file type “Other” when you re-submit your manuscript. This document is for internal journal use only and will not be published if your article is accepted. The requested information will help us to assess whether your submission complies with PLOS’s policies and adheres to applicable reporting standards. Note that your manuscript may be rejected if you provide incomplete or inadequate responses to the checklist questions and that changing the ‘Section/Category’ of your article does not affect this requirement.

**Response**

From the checklist:

- If you did not obtain ethical approval, please explain why this was not required below.

The Ministry of Health of Uganda gave the directive and approval to investigate this Sudan virus Disease outbreak. In agreement with the International Guidelines for Ethical Review of Epidemiological Studies by the Council for International Organizations of Medical Sciences (1991) (39) and the Office of the Associate Director for Science, CDC/Uganda, it was determined that this activity was not human subject research and that its primary intent was public health practice or disease control activity (specifically, epidemic or endemic disease control activity). Verbal informed consent was obtained from the participants before the start of each interview. Parental/legal guardian verbal informed consent was obtained on behalf of all the children before the start of each interview since they were aged less than 10 years. The authors sought permission to conduct the study from the Ministry of Health, and Uganda Public Health Fellowship program. Data which was collected did not contain individual personal identifiers as a way of ensuring confidentiality. This activity was reviewed by CDC and was conducted consistent with applicable federal law and CDC policy. §

§See e.g., 45 C.F.R. part 46, 21 C.F.R. part 56; 42 U.S.C. §241(d); 5 U.S.C. §552a; 44 U.S.C. §3501 et seq

This has been added to lines 148 to 162, page 8

1. If you are reporting a retrospective study of medical records or archived samples, please report in the Methods section:
i. the day, month and year when the data were accessed for research purposes
ii. whether authors had access to information that could identify individual participants during or after data collection

**Response**

The study was determined to be non-research (Page 8, lines 148-154). We obtained de-identified data for this study on 15/01/2023 (Page 8, lines 158-159)

**Comment 4**

Your study includes live participants, please update your Ethics Statement with confirmation that formal consent was obtained (must state whether verbal/written).

**Response**

Verbal informed consent was obtained from the participants before the start of each interview. Parental/legal guardian verbal informed consent was obtained on behalf of all the children before the start of each interview since they were aged less than 10 years. This has been added to line 154-156, page 8
