# Supplementary material for: Use of the Chainchecker application: Uganda’s experience during the 2022 Sudan Virus Disease outbreak
Source: PLOS Glob Public Health. 2025 Apr 21;5(4):e0004352. doi: 10.1371/journal.pgph.0004352 (PMC12011214; doi:10.1371/journal.pgph.0004352)
Supplement: Attachment — Submitted filename: Response to reviewers.docx [file pgph.0004352.s003.docx]

Dear Dr Flavio Finger, we sincerely appreciate the reviewers’ thorough evaluation of our manuscript, ‘Use of the Chainchecker application: Uganda’s experience during the 2022 Sudan Virus Disease outbreak’. We are grateful for their insightful and constructive comments, which have helped us improve the quality and clarity of our work.

Below, we respond to the reviewers’ comments and indicate changes made to the tracked copy of the manuscript.

**Editor comments**

Journal Requirements:

1. Please provide additional details regarding participant consent. In the ethics statement you have stated that verbal informed consent was obtained from participants before the start of each interview. Please can you clarify if the participants were consenting to participation in epidemiological surveillance or this specific research study.

Please can you also clarify whether the interviews took place as part of epidemiological surveillance and whether the data from these were anonymised prior to you accessing it? Or were the interviews conducted by you directly as part of this study.

**Response**

Thank you. Verbal informed consent was obtained from the participants before the start of each interview as part of the epidemiological surveillance activities for EBOD; the interviews weren’t undertaken as part of this specific study. Data (accessed on 15/01/2023) did not contain individual personal identifiers as a way of ensuring confidentiality. This has been added to the ethics statement, lines 164-165 and 168-169 of the tracked copy of the manuscript.

2. Please provide separate figure files in .tif or .eps format.

For more information about figure files please see our guidelines:
<https://journals.plos.org/globalpublichealth/s/figures> 
<https://journals.plos.org/globalpublichealth/s/figures#loc-file-requirements>

**Response**

Thank you. We have uploaded the figures in .tif using the PACE software.

3. Please review your reference list to ensure that it is complete and correct. If you have cited papers that have been retracted, please include the rationale for doing so in the manuscript text, or remove these references and replace them with relevant current references. Any changes to the reference list should be mentioned in the rebuttal letter that accompanies your revised manuscript. If you need to cite a retracted article, indicate the article’s retracted status in the References list and also include a citation and full reference for the retraction notice.

**Response**

We have reviewed and updated the reference list to ensure that all cited papers are complete, accurate, and up-to-date. We have included new references to expand the discussion section: references [14] line 246, and [16, 17] lines 257 in the tracked copy of the manuscript to expand the discussion section. We also included the references citations on the following lines as responses to reviewer comments: lines 125, 126, page 7.

**Reviewer #1:**

Overall, this is a solid paper on a very useful application, with clear use cases. This tool will make epidemic investigations easier and faster, and the combination of genomic and epidemiological data in particular is a very important innovation.

 I have some minor comments

**Comments**

 • It sounds like Chainchecker is used on a computer with CSV data uploaded – are there any plans to make it a phone app which you can input data directly into?

**Response**

Thank you, this is an interesting idea! As of now, we do not have plans to convert chain checker into a phone app - mainly due to the difficulty in updating the CSV data on a phone. But it is something worthwhile to consider, especially since most outbreaks happen in resource-limited settings.

**Comments**
 • An example of what would go in the “source” field would be helpful – is that a location, or a known infecting case?

**Response**

This is the source of infection (known infecting case). We’ve also updated the tool so that users could add in hypothetical sources, such as an animal (ie - bat), or another individual (the lines connecting hypothetical cases to true cases is dashed to indicate uncertainty). This has been added to page 5, line 81-83.

**Comment**
 • There should be a citation for ape on line 120

**Response**

Thank you, we have included citations on line 125, page 7 of the tracked version. And for nucleotide substitution models on line 126.

**Comment**
 • I’m not clear on how other nucleotide substitution models would be included, given that it sounds like the raw distance is what’s used? Some more detail on how genetic distance is calculated would be helpful.

**Response**

Thank you - great question. We use the dist.dna function in R to generate a genetic distance matrix (the matrix compares all genomes to each other). Initially, we calculated genetic distance using a nucleotide substitution model included in the dist.dna function (similar to how phylogenetic trees are built), but found the values hard to interpret. Rather, we found it easier to use raw nucleotide differences (also included as an option in the dist.dna function) when comparing genomes between individuals - ie, integer nucleotide values (0-5 nucleotides). Genetic distance is calculated using dist.dna (model="raw") and the resulting values are multiplied by the total number of analyzed nucleic acids, which results in integer differences between genomes.

**Comment**
 • What software is used to align sequences?

Response

We used mafft (v7.490) - I have added this to line 123, page 6 of the tracked version of the manuscript.

**Comment**
 • How are indels taken into account in terms of genetic distance? Ebola viruses have intergenic regions, and so indels may be real.

**Response**

Indels are not currently included when calculating genetic distance. Ebola viruses do have intergenic regions, but they do not usually have large indels (or really any significant) indels in the intergenic regions. Also, the genetic analysis is not performed in the ChainChecker tool. The user is free to use any tool to calculate genetic distance, including one that is more lenient with indels.

**Comment**
 • How do you exclude poorly sequenced data? Or in general is there any quality control in the application itself, or are you relying on good genomic data and associated metadata going in? Without any quality control, there may be transmissions ruled out that are real epidemiological connections.

**Response**

Good question! The ChainChecker application does not perform any of the genetic analysis - the user is responsible for excluding poor sequence data, building fastas, making alignments and calculating genetic distance separate from Chainckecker. However, we have released the code that we used for building the genetic distance matrix on github (<https://github.com/evk3/ChainChecker>).

The reviewer raises an important point - poor quality or limited sequence coverage will limit the connections and interpretations that one can make with Chainchecker.

We’ve also added a component to ChainChecker that allows users to modify the epidemiological metadata in the tool (without having to re-load a corrected input file). This allows for more active data pruning and correction within the tool (and one can download the corrected line listing, too).

**Comment**
 • Does the epidemiological connection have to be direct? Or does it just mean they are part of the same chain?

**Response**

No, the epidemiological connections do not have to be direct for individuals to be plotted in the tool. In fact, that’s one of the cool things about the tool - one can visualize “broken” epi connections, and connect folks to existing chains using the genetic data.

**Comment**
 • NB The figures are very low resolution so it’s hard to assess them.

**Response**

Thank you for the feedback. We have edited the figures to improve their resolution.

**Reviewer #2:**

This is a very interesting paper explaining how an innovative application can help to determine epidemiological link between Ebola cases which is the key to adequately response and control an Ebola Virus Disease outbreak.

However, here are a few comments :

1. How does the health status of a confirmed case (dry phase, humid phase or dead body) impact the calculation of the incubation knowing that the risk to develop the disease may not be the same when contact occurs at those different phases of the disease?

Response

The Chainchecker application calculates exposure windows for cases using user-defined incubation periods (minimum and maximum durations that account for the health status of the case), dates of symptom onset, and periods of infectiousness. While the application does not directly adjust for variations in risk associated with different phases of a confirmed case (e.g., dry phase, humid phase, or post-mortem exposure), these phases can influence the likelihood of transmission. This variability is accounted for by using the minimum and maximum incubation periods(informed by previous outbreaks) in the calculations. Additionally, contacts of cases( in the dry phase, humid phase, or following post-mortem exposure) were isolated for 21 days, during which they were observed for signs and symptoms of EBOD. This has been added to page 5, lines 94-95)

2. Figure 1a and 1b are not readable.

Response

Thank you for the feedback. These have been edited to be more readable.

3. To what extent Chainchecker can be used in different diseases such Mpox, in case of sustained human-to-human transmission?

Response
 Chainchecker is designed as a versatile tool that can be adapted for use in tracking transmission dynamics of various infectious diseases, including Mpox, during sustained human-to-human transmission. The tool relies on user-defined parameters, such as incubation periods, dates of symptom onset, and periods of infectiousness, which can be customized to reflect the epidemiological characteristics of any disease.

For Mpox, the incubation period, modes of transmission (e.g., direct contact with lesions, bodily fluids, or contaminated materials), and the infectious period would need to be accurately defined and incorporated into the application's configuration. By doing so, Chainchecker could effectively calculate exposure windows and assist in verifying transmission chains.

The utility of Chainchecker in other infectious diseases including Mpox has been added to 293-297, page 14 of the tracked version of the manuscript.

**Reviewer #3**:

The authors present a description of the use of the application ChainChecker for a recent disease outbreak in Uganda. Whilst the application has been previously described, the authors present an updated version and, of particular note, validate its use with genomic data. This highlights the benefits of clear visualisations for real time outbreak response. Though, the current work suffers from one of the same issues as the first tool: the tool captures uncertainty only through a range on the exposure window rather than in other areas such as the reported dates. However, as shown, coupled with other data sources and expertise of those conducting the outbreak response, it can contribute to the suite of activities.

Major notes: It would be good to expand on the limitation of uncertainty in the discussion ie. that it is only captured through the exposure window.

Response

Thank you, we have included this limitation ‘Although ChainChecker is a valuable tool for real-time outbreak response, its ability to capture uncertainty is limited primarily to exposure windows defined by minimum and maximum incubation periods. It does not account for uncertainties in reported dates of symptom onset, exposure, or infectious periods, which may arise from recall bias or incomplete data. Future enhancements could address these limitations by incorporating probabilistic modeling or confidence intervals for key inputs and integrating additional metadata. Despite these constraints, ChainChecker remains a useful complement to other epidemiological and genomic data, enabling more effective outbreak response.’ on page lines 273-280, page 13.

Minor notes: the references do not appear to line up correctly. Often reference 8 is mentioned for chainchecker but this refers to a different outbreak of Hepatitis rather than reference 7 which desciribes the original chainchecker tool.

Response

This has been corrected, thank you.

**Reviewer #4:**

In the title, change Ebola virus disease into Sudan virus disease, as this latter is most cited in the manuscript.

Agreed! we have made this correction.

Introduction

1) Maybe authors can use standard abbreviations such as EVD instead of EBOD

Response

Thank you for this comment. In line with the most recent updates to filovirus nomenclature, the standardized abbreviation for Ebola Disease is EBOD, as it encompasses a broader classification that includes several specific variants: 1D60.00 Bundibugyo virus disease (BVD), 1D60.01 Ebola virus disease (EVD), 1D60.02 Sudan virus disease (SVD), 1D60.03 Atypical Ebola disease, 1D60.0Y Other specified Ebola disease, and 1D60.0Z Ebola disease, virus unspecified. This updated terminology aims to provide a unified framework for categorizing Ebola Disease. <https://pmc.ncbi.nlm.nih.gov/articles/PMC6637750/#SD1>.

2) Authors could lay more on inconsistencies created by the use of previous data reporting system to show the associated gaps, then the need to fully address these through the chainchecker application.

Response

Thank you for your feedback. We have included language describing inconsistencies in data management from previous outbreaks at lines 54-58. We also include an example in figure 2, panel B of hand-drawn chains initially used in this outbreak and previous outbreaks.

Methods

1) Line 87-89: how maximum and minimum incubation periods were they set for all confirmed-cases? Were they fixed or varying from one case to another? How did the authors determine them throughout the study? Can authors can lay more on that.

Response

Thank you - the maximum and minimum incubation periods are integer values that are set by the user. These values are universally applied to all samples in the analysis and define a range in time before the symptom onset date. We found that highlighting an incubation range is useful when trying to establish whether epidemiological connections are biologically plausible. For Ebola disease, there have been multiple publications that question the 2 day minimum incubation period, as such the minimum default was set at 4 days. However, as mentioned previously, the default minimum and maximum incubation period can be adjusted by the user.

2) Line 95-97 is unclear. Would authors meant “stacked vertically”? That would more sense for the full sentence…

Response

Thank you! We have corrected this typo.

3) Line 142: correct this “de-identified data outbreak data”

Response

Thank you! We have corrected this typo, lines 173-174.

4) Lines 144-146: look like results

Response

Thank you! Good idea, we moved these sentences to the Results section, lines 171-177.

5) Ethical considerations section can be summarized.

Response

Thank you, we have kept the text as written because it is important to demonstrate that a thorough review was conducted both at the CDC and in Uganda.

Discussion

1) The discussion is short and not consistent enough to support findings of this study.

Response

Thank you, we have expanded the discussion and included additional references to support the statements.

2) Provide an in-depth discussion of key findings

Response

Thank you, we have added content to the discussion section

3) Provide study limitations

Response

Thank you, we have added this to lines 273-280, page 13.

4) Is it possible to extend the use of this tool to other diseases, such as mpox? (Lay a little bit more on that, not only one or two sentences).

Response

Thank you, we have included this at lines 292-297.
